# Prevalence of Inherited Hemoglobin Disorders and Relationships with Anemia and Micronutrient Status among Children in Yaoundé and Douala, Cameroon

**DOI:** 10.3390/nu9070693

**Published:** 2017-07-03

**Authors:** Reina Engle-Stone, Thomas N. Williams, Martin Nankap, Alex Ndjebayi, Marie-Madeleine Gimou, Yannick Oyono, Ann Tarini, Kenneth H. Brown, Ralph Green

**Affiliations:** 1 Department of Nutrition, University of California, Davis, CA 95616, USA; khbrown@ucdavis.edu or Ken.Brown@gatesfoundation.org; 2 KEMRI/Wellcome Trust Research Programme, Kilifi, Kenya; tom.williams@imperial.ac.uk; 3 Helen Keller International, Cameroon, BP 14227 Yaoundé, Cameroon; nankapm@gmail.com (M.N.); andjebayi@hki.org (A.N.); tariniann@gmail.com (A.T.); 4 Centre Pasteur du Cameroun, BP 1274 Yaoundé, Cameroon; oyonoyannick@gmail.com; 5 Bill & Melinda Gates Foundation, Seattle, WA 98102, USA; 6 Department of Medical Pathology and Laboratory Medicine, UC Davis Medical Center, Sacramento, CA 95817, USA; ralph.green@ucdmc.ucdavis.edu

**Keywords:** anemia, hemoglobinopathy, iron, children, thalassemia, sickle cell

## Abstract

Information on the etiology of anemia is necessary to design effective anemia control programs. Our objective was to measure the prevalence of inherited hemoglobin disorders (IHD) in a representative sample of children in urban Cameroon, and examine the relationships between IHD and anemia. In a cluster survey of children 12–59 months of age (*n* = 291) in Yaoundé and Douala, we assessed hemoglobin (Hb), malaria infection, and plasma indicators of inflammation and micronutrient status. Hb S was detected by HPLC, and α^+^thalassemia (3.7 kb deletions) by PCR. Anemia (Hb < 110 g/L), inflammation, and malaria were present in 45%, 46%, and 8% of children. A total of 13.7% of children had HbAS, 1.6% had HbSS, and 30.6% and 3.1% had heterozygous and homozygous α^+^thalassemia. The prevalence of anemia was greater among HbAS compared to HbAA children (60.3 vs. 42.0%, *p* = 0.038), although mean Hb concentrations did not differ, *p* = 0.38). Hb and anemia prevalence did not differ among children with or without single gene deletion α^+^thalassemia. In multi-variable models, anemia was independently predicted by HbAS, HbSS, malaria, iron deficiency (ID; inflammation-adjusted ferritin <12 µg/L), higher C-reactive protein, lower plasma folate, and younger age. Elevated soluble transferrin receptor concentration (>8.3 mg/L) was associated with younger age, malaria, greater mean reticulocyte counts, inflammation, HbSS genotype, and ID. IHD are prevalent but contribute modestly to anemia among children in urban Cameroon.

## 1. Introduction

Anemia affects an estimated 600 million children globally and, when severe, increases the risk of morbidity and mortality [1,2]. Many conditions can lead to anemia in the pediatric age group, including deficiencies of iron, vitamin A, and B vitamins. Malaria and helminth infections, where these conditions are present, and inherited hemoglobin disorders (IHD), such as sickle cell disease (SCD) or thalassemias, contribute to the burden of anemia [3,4]. Although the magnitude of iron-deficiency anemia (IDA) globally is uncertain and likely varies by population, iron deficiency (ID) is considered the most common cause of anemia [5]. For this reason, national policy in many countries recommends presumptive treatment of anemia with iron supplements. However, iron supplementation of anemic individuals without ID is ineffective and wastes resources. Moreover, there is concern that iron supplementation of iron-replete individuals, particularly in settings with a high infectious disease burden, may be harmful [6,7]. Thus, information on the likely etiology of anemia in particular settings is necessary for the development of effective and efficient anemia-control programs. In addition, some nutrition programs, such as large-scale fortification of staple foods with iron, still rely on hemoglobin (Hb) as an indirect indicator of program efficacy due to relative low cost and ease of measurement. Estimates of the proportion of anemia attributable to deficiencies of iron and other micronutrients also provide insight into the extent to which Hb would be expected to respond to a nutrition intervention program. 

IHD include structural ß-globin chain hemoglobin variants such as hemoglobin C, E, and S, and α- and ß-thalassemias (variants or deletions in the α- and ß-globin chains of hemoglobin, respectively, giving rise to quantitative imbalances between α- and ß-globin chains), and can lead to anemia via reduced or abnormal hemoglobin synthesis [8]. The two main disorders observed in sub-Saharan Africa are HbS and α^+^thalassemia; these are thought to be common due to genetic selection because of the protection these conditions provide against falciparum malaria morbidity and mortality [9,10], although this protection appears to be lost in the case of co-inheritance of Hb S trait and an α-globin deletion [11]. Globally, an estimated 305,800 children are born with sickle cell disease (HbSS) annually, with almost 80% of this burden in sub-Saharan Africa [12]. An estimated 20.7% of the population either has heterozygous or homozygous α^+^thalassemia (one and 2 alpha-globin gene deletions, respectively), with an estimated 41.2% of these occurring in Africa and 44.6% in South-East Asia [13]. Country-specific data on the prevalence of these conditions, as well as the extent to which they contribute to anemia [14], are necessary to construct public health programs that address modifiable causes of anemia. Although sickle cell disease affects a relatively small percentage of children, the clinical symptoms are much more severe than those of α^+^thalassemia; however, it is possible that clinically mild or asymptomatic conditions, such as HbAS or α^+^thalassemia, may explain some proportion of low Hb concentrations at the population level. Interactions between IHD and nutritional status are also possible; for example, it has been hypothesized that α^+^thalassemia may be protective against ID by increasing iron absorption, although some analyses have not supported this hypothesis [15,16]. 

Soluble transferrin receptor (sTfR) concentrations in plasma rise in response to ID, reflecting increased transferrin receptor expression in developing erythrocytes [17]. However, increased erythropoietic activity, for example in response to malaria-induced hemolytic anemia, may also increase sTfR concentrations in plasma [18,19]. Thus, concerns have been raised that conditions such as hemoglobinopathies and malaria infection will confound interpretation of sTfR concentrations for the assessment of ID in settings such as sub-Saharan Africa [19]. 

The primary objective of this study was to measure the prevalence of inherited hemoglobin disorders in a representative sample of young children in the two major cities of Cameroon, and examine the relationship between IHD and anemia. In particular, we were interested in examining carrier status (HbAS or α^+^thalassemia, α–/αα genotype), which may be clinically asymptomatic and thus would not prevent children from participating in a survey of apparently healthy children, but still potentially contribute to shifts in indicators of interest at the population level. We also used multivariable models of factors associated with Hb concentration and anemia to examine the associations between IHD and anemia independent of other covariates. In addition, because IHD may be associated with inflammation and risk of disease (such as malaria), we conducted an exploratory analysis to examine relationships between IHD and indicators of micronutrient status and inflammation. Finally, we used available data on iron status and non-nutritional causes of increased erythropoiesis to examine the use of sTfR as an indicator of iron status. 

## 2. Materials and Methods 

### 2.1. Study Design and Participants

The study design and methods have been described in detail elsewhere [20,21,22]. The primary objective of the study was to assess the change in micronutrient status following implementation of a large-scale food fortification program. The survey was designed to represent children 12–59 months of age and their female caregivers in Yaoundé and Douala, the two largest metropolitan areas in Cameroon. 

Within each city, 15 clusters were selected using proportionate-to-population size sampling of enumeration areas defined by the Cameroon census bureau (BUCREP). For each cluster, households were sampled by selecting a random start point (walking in a randomly selected direction from the center to the edge of the cluster, and randomly selecting a house along that line as a starting point) and systematically sampling adjacent households. The target sample size (10 households per cluster; 300 households total) was based on expected changes in micronutrient status indicators post-fortification and was estimated to be sufficient to measure a prevalence of hemoglobin disorders of 25% with 5% precision and to detect an effect size of 0.31 for the difference in Hb concentration among children with and without hemoglobin disorders, assuming a design effect of 2.

Households were eligible to participate if they included a child 12–59 months of age and the child’s primary female caregiver (15–49 years of age), and if the selected woman and child had lived in the household for at least one month. Exclusion criteria were self-reported diarrhea with dehydration, severe fever, or other severe illness experienced by the woman or child in the 3 days prior to data collection. 

Women provided informed oral consent for themselves and their children to participate. The study was approved by the Institutional Review Board of the University of California, Davis (Protocol #364876 for overall survey and #553562 for hemoglobinopathy objective), and the Ministry of Public Health of Cameroon (during a period of reorganization during which the National Ethics Committee was not available to review applications). 

### 2.2. Data Collection

Interviewers administered questionnaires to the female respondent to obtain information on participant demographic and socio-economic characteristics. The respondent’s (self-reported) ethnic group was recorded as a proxy for that of the child. Participant height/length and weight were measured by stadiometer (for children ≥ 2 years of age and for women)/length board (for children < 2 years) and electronic scale, respectively. 

Venous blood samples were collected at a central location in each cluster, with appropriate precautions to protect light-sensitive analytes and to minimize external or internal (e.g., from erythrocytes) zinc contamination [23]. Blood was collected into trace-element free tubes containing lithium heparin (Sarstedt, Nümbrecht, Germany); tubes were placed in a cooler with ice packs prior to centrifugation to obtain plasma. A second blood sample was collected (without removing the needle) into an EDTA-coated tube for assessment of malaria infection, hemoglobin concentration, and reticulocyte counts using whole blood. 

Malaria was assessed using a rapid diagnostic test (SD Bioline Malaria Ag Pf/Pan, Standard Diagnostics; Gyeonggi-do, Republic of Korea) and hemoglobin was measured by portable photometer (Hemocue, Ängelholm, Sweden). Individuals with positive tests were provided with treatment and referred to the nearest health center. The remaining aliquot of whole blood was transferred to the Centre Pasteur of Cameroon for reticulocyte analysis on the day of collection (for samples collected in Yaoundé) or on the following day, but within 24 h of collection (for samples collected in Douala). 

Plasma samples were aliquoted into cryotubes within a portable plastic “hood” (to minimize light exposure and contamination with dust). Samples of the buffy coat and red cells were retained for measurement of α-globin genotype and hemoglobin variants (e.g., Hb S), respectively. Plasma and red and white cell samples were stored in a cooler until being transferred to a −20 °C freezer at the end of the day. 

Frozen samples were shipped on dry ice to Germany for analysis of plasma proteins at the VitA-Iron lab, and to the United States for analysis of plasma folate and B12 at the Western Human Nutrition Research Center, plasma zinc at the Children’s Hospital Oakland Research Institute, and Hb variants at the UC Davis Medical Center. Buffy coat aliquots were shipped to the KEMRI/Wellcome Trust Research Programme, Kilifi, Kenya for α^+^thalassemia genotype assessment.

### 2.3. Laboratory Analyses

Reticulocytes were measured by fluorescence detection (PENTRA 120 analyzer, ABX DIAGNOSTICS) after staining cells with Thiazole Orange dye (Becton Dickinson, San Jose, CA, USA). Level 2 (normal) Internal Quality Control was conducted daily prior to analysis, using ABX MINOTROL RETIC (HORIBA ABX SAS, Montpellier, France).

Plasma C-reactive protein (CRP) and α_1_-acid glycoprotein (AGP) (markers of inflammation), ferritin and soluble transferrin receptor (sTfR) (markers of iron status), and retinol-binding protein (marker of vitamin A status) were measured by ELISA [24]. Plasma folate and vitamin B-12 were measured using a SimulTRAC-SNB radioimmunoassay kit (57Co/125I) (MP Diagnostics) [25]. Plasma zinc was measured by inductively coupled plasma-optical emission spectrometry (ICP-OES) [26]. 

Hemoglobin variants were assessed by HPLC using an ultra Resolution Variants Analyzer (Trinity Biotech, Bray, Ireland). Typing for α^+^thalassemia genotyping was conducted by PCR analysis as described in detail previously [27]. Heterozygous α^+^thalassemia indicates deletion of a single α-globin gene, and homozygous α^+^thalassemia indicates deletion of two α-globin genes. β-thalassemia was not investigated because it is considered to be rare in sub-Saharan Africa.

### 2.4. Data Analysis

Data were entered in Access and analyzed in SAS v9.4 (SAS Institute, Cary, NC, USA). Survey procedures with appropriate survey weights were used to calculate descriptive statistics. Values for children with and without the selected Hb disorders were estimated by domain analysis. 

Both study sites are located at an altitude <1000 m; therefore, Hb was not adjusted for altitude. We adjusted plasma concentration of ferritin, RBP, and zinc for inflammation using a regression approach. For each biomarker, we constructed a regression equation with the biomarker as the dependent variable and CRP and AGP as independent variables (with interactions and square terms retained where these were significant) [28,29]. For all observations, we then used the regression coefficients from these equations to adjust the biomarker concentrations to values equivalent to the 10th percentile of the CRP and AGP concentrations [29] among the “healthy reference group” of children (those with CRP < 5 mg/L and AGP < 1 g/L); the reference concentrations selected using this approach were CRP = 0.14 g/L and AGP = 0.52 g/L. Ferritin and RBP were adjusted for CRP only, because AGP was not a significant predictor of either ferritin or RBP when included in a regression equation along with CRP as an independent variable; however, plasma zinc concentration was adjusted for both CRP and AGP. Total body iron stores were estimated by applying Cook’s formula using sTfR and regression-adjusted ferritin [30]. Iron deficiency was defined as regression-adjusted ferritin concentrations <12 µg/L or estimated body iron stores < 0 mg/kg, vitamin A deficiency was defined as regression-adjusted plasma RBP concentrations <0.83 µmol/L [31], and low plasma zinc was defined as regression-adjusted plasma zinc <65 µg/dL for AM samples and <57 µg/dL for PM samples [23]. Anemia and severe anemia were defined as Hb < 110 g/L and < 70 g/L, respectively [32]. 

We used linear and logistic regression (SAS proc surveyreg and proc surveylogistic), as appropriate, to compare characteristics of children with HbAS vs. HbAA genotype, children with heterozygous α^+^thalassemia compared with no α^+^thalassemia gene deletion, and children with elevated vs. normal sTfR. Because the number of cases of HbSS (*n* = 5) and homozygous α^+^thalassemia (*n* = 9) was small, we excluded these from bivariate comparisons by genotype, and focused instead on assessing the effect of carrier status for Hb S or heterozygous α^+^thalassemia. However, HbSS and homozygous α^+^thalassemia were included in the bivariate and multivariate models to predict Hb concentration and anemia, as described below. 

We used SAS survey procedures for linear and logistic regression, respectively (proc surveyreg; proc surveylogistic), to examine predictors of Hb concentration and anemia. We first examined bivariate relationships between each outcome and each independent variable. Next, to examine independent predictors of Hb and anemia, we combined all independent variables into the same model and sequentially removed non-significant (*p* > 0.05) variables until reaching a final model in which all variables were statistically significant. Due to the sample size, we did not examine any interactions other than that between CRP and AGP, which was not significant. Due to the complexity of correcting biomarkers of iron, vitamin A, and zinc status for inflammation, these adjusted values were dichotomized to define deficiency for each nutrient (as defined above). Because folate and B12 were not related to inflammation, and because folate and B12 concentrations below the cutoffs for deficiency (folate < 10 nmol/L and B12 < 221 pmol/L) were rare, these variables were included as continuous variables. Regression diagnostics (normality of residuals and outlier exclusion using proc surveyreg, and leverage and collinearity diagnostics as assessed with proc reg) were acceptable for the model containing all covariates and the final, reduced model.

## 3. Results

### 3.1. Household and Participant Characteristics

Details of the survey recruitment and response rate have been presented elsewhere [20,21]. Thirty-four different ethnic groups were represented by the mothers (not including subgroups within these ethnic groups), in addition to a small proportion (<2%) who reported being of mixed ethnicity, and 2 mothers of non-Cameroonian descent. The largest ethnic group represented was Bamiléké (33% of participants). Other major groups included Ewondo (11%), Bassa (8%), Douala (7%), and Bamenda (6%).

In this urban population, the prevalence of stunting (16%) and wasting (1.4%) was low relative to the national average (33% stunted and 5.6% wasted in 2011[33]). Prevalence of current or recent malaria infection (8.0%) was also relatively low, but inflammation was common (46%) (Table 1). Malaria was marginally associated with inflammation (defined as elevated CRP and/or AGP, Rao-Scott chi-square *P* = 0.07). The majority of households had access to running water and improved toilet facilities: running water (inside or outside the home) was available in 66% of households, while 26% relied on wells, and 8% on other sources. Household toilet facilities included modern toilets (27%), ventilated improved pit latrines (29%) and pit latrines with (30%) and without (13%) covers. 

Anemia was present among 45% of children, but only 1% were severely anemic. Depending on the iron status indicator used, estimates of iron deficiency ranged from 13% (inflammation-adjusted plasma ferritin concentrations < 12 µg/L) to 25% (plasma sTfR > 8.3 mg/L). Deficiencies of vitamin A and zinc were prevalent (13.5 and 23.2%, respectively, following adjustment for inflammation), but almost no children had low plasma folate or vitamin B-12 concentrations. 

### 3.2. Prevalence of Inherited Hemoglobin Disorders

Overall, 43% of children had HbS or α^+^thalassemia; the HbS allele was present in 15% of children and 34% of children had at least one α-globin deletion (Table 2). Although the study population included clinically asymptomatic children, 5 children were diagnosed as having sickle cell disease (HbSS), and 9 children were identified as having homozygous α^+^thalassemia. Approximately 4% of children (*n* = 12) had co-inherited the HbAS genotype and heterozygous α^+^thalassemia; 3 children had two gene deletion α^+^thalassemia and HbAS genotype, and 4 of the 5 children with HbSS also had heterozygous α^+^thalassemia.

Children whose mothers reported being from the Bamiléké ethnic group (the most common group in the survey population) had a lower prevalence of α-globin deletions and Hb S compared with the other ethnic groups combined (any α-globin deletion: 20% vs. 41%, *p* < 0.01; HbS: 8.5% vs. 18.3%, *p* = 0.010). However, the prevalence of malaria and anemia did not differ between children of Bamiléké mothers compared with other ethnic groups (*p* > 0.3), with or without stratifying by child sex. 

### 3.3. Relationships between Inherited Hemoglobin Disorders and Anemia

**Figure 1:**
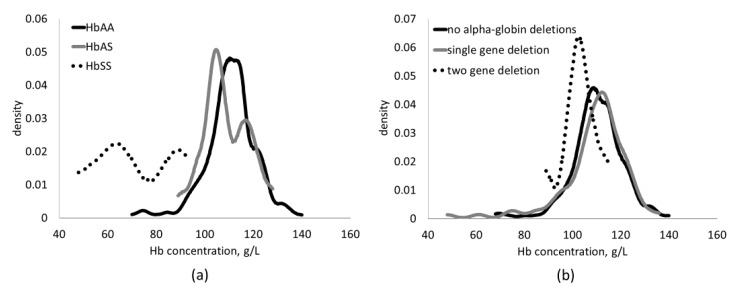
Kernel density plots illustrating the distribution of hemoglobin concentrations among: (**a**) children with HbAA (*n* = 250), HbAS (*n* = 40), and Hb SS (*n* = 5) genotypes; and (**b**) among children with none (*n* = 190; α α/α α), one (*n* = 89; α–/α α), or two (*n* = 9; α–/α–) alpha-globin deletions.

Compared to prevalence of anemia among HbAA children (and excluding children with HbSS and homozygous α^+^thalassemia), the prevalence of anemia was greater among HbAS children (mean ± SE: 60.3 ± 7.6% vs. 42.0 ± 3.3%, *p* = 0.038) (Figure 1). However, mean hemoglobin concentrations did not differ (mean ± SE: 109 ± 1 and 111 ± 1 g/L; *p* = 0.38) for HbAS and HbAA, respectively. Mean hemoglobin concentrations (mean ± SE: 110 ± 1 and 109 ± 1; *p* = 0.73) and prevalence of anemia (% ± SE: 46.3% ± 3.1 vs. 42.6% ± 6.3; *p* = 0.39) did not differ among children with no α-globin deletions and children with heterozygous α^+^thalassemia, respectively (and excluding children with HbSS and homozygous α^+^thalassemia). 

### 3.4. Predictors of Anemia

In bivariate models, Hb concentration was negatively associated with HbSS genotype, homozygous α^+^thalassemia, malaria, CRP, AGP, vitamin A deficiency (low inflammation-adjusted RBP), and iron deficiency (low inflammation-adjusted ferritin) and positively associated with plasma folate and vitamin B-12 concentrations (Table 3). There were also marginally significant positive associations between Hb and age (*p* = 0.088) and female sex (*p* = 0.081). Relationships between Hb concentration and age, HbSS genotype, homozygous α^+^thalassemia, iron deficiency, vitamin A deficiency, and plasma AGP and vitamin B-12 concentrations remained significant in the multivariable model.

Factors associated with anemia in bivariate logistic regression models were younger age, HbAS, HbSS, homozygous α^+^thalassemia, malaria, vitamin A deficiency, iron deficiency, greater plasma CRP and AGP concentrations and lower plasma folate and lower vitamin B-12 concentrations. In the multivariable model, the factors that remained independently associated with anemia were age, HbSS genotype, malaria, vitamin A deficiency, iron deficiency, and plasma CRP concentrations, although marginally significant relationships were observed for homozygous α^+^thalassemia (*p* = 0.11) and plasma vitamin B-12 concentrations (*p* = 0.08).

### 3.5. Relationships between Inherited Hemoglobin Disorders and Micronutrient Status

Compared to HbAA children, HbAS children were more likely to have higher inflammation-adjusted ferritin concentrations (36.8 vs. 30.3 µg/L, *p* = 0.040). In addition, adjusted plasma RBP concentrations were marginally higher (1.10 vs. 1.05 µmol/L; *p* = 0.089), and low adjusted plasma RBP concentrations were marginally less frequent (2.5 vs. 14.3%; *p* = 0.057), among HbAS children compared with HbAA children. There were no other statistically significant differences between children with the HbAA vs. HbAS genotype with regard to age, inflammation markers, malaria, or indicators of micronutrient status.

Compared to children with no α-globin deletions (and excluding children with combined HbSS and homozygous α^+^thalassemia), children with heterozygous α^+^thalassemia had higher reticulocyte counts (0.55 vs. 0.42 × 10^5^ cells/µL; *p* = 0.035) and lower prevalence of inflammation (35.8 vs. 49.9%; *p* = 0.008). In addition, plasma AGP concentrations were marginally lower (0.92 vs. 0.99 g/L; *p* = 0.08), sTfR concentrations were marginally higher (8.36 vs. 8.10 mg/L; *p* = 0.06), and the prevalence of low inflammation-adjusted body iron stores was marginally higher (17.5% vs. 8.0%; *p* = 0.06) among children with heterozygous α^+^thalassemia compared to those with no α-globin deletions. There were no other statistically significant differences in measured characteristics, including anemia and micronutrient status, between children with and without a single α-globin deletion.

### 3.6. Interpretation of Soluble Transferrin Receptor

Compared to children with sTfR < 8.3 mg/L, children with elevated sTfR were younger and more likely to be male and have malaria, inflammation (elevated CRP and/or AGP), iron deficiency (defined either as inflammation-adjusted ferritin < 12 µg/L or BIS < 0 mg/kg), and the HbSS genotype (Table 4). In addition, children with elevated sTfR had greater mean reticulocyte counts and were more likely to have reticulocyte counts above 1.5 × 10^5^ cells/µL. There were no differences in concentrations of indicators of vitamin A, zinc, folate, or vitamin B-12 status, or α^+^-thalassemia status between children with and without elevated sTfR (although, as noted above, mean sTfR concentrations tended to be higher among children with a single α-globin deletion).

Of 76 children with elevated sTfR, 22 had low inflammation-adjusted ferritin and 19 had malaria, a hemoglobinopathy (HbSS) or homozygous α^+^thalassemia, and/or elevated reticulocytes (i.e., causes or evidence of increased erythropoiesis); 3 children had results consistent with both iron deficiency (as assessed by adjusted ferritin) and increased erythropoiesis (as assessed by reticulocyte number).

## 4. Discussion

We observed that IHD were prevalent among young, asymptomatic children in Yaoundé and Doula, Cameroon: 43% of children had either the Hb S trait or at least one α-globin deletion. Mean Hb concentrations did not differ among Hb S carriers compared with HbAA children; but, due to a shift in the lower tail of the Hb distribution, HbAS children were more likely to be anemic compared with HbAA children in both bivariate comparisons and multi-variable models. Homozygous, but not heterozygous, α^+^thalassemia was also associated with both lower mean Hb concentration and greater prevalence of anemia. These results suggest that clinically asymptomatic IHD, such as α^+^thalassemia, may contribute to anemia among children in this setting, although the absolute difference in anemia prevalence between children with and without IHD was modest compared to the total prevalence of anemia.

Four of the 5 children with HbSS also had heterozygous α^+^thalassemia. Co-inheritance of these IHD is associated with a less severe phenotype [34], which may explain why the children were clinically asymptomatic and thus identified as eligible to participate in the survey (“apparently healthy”). We also observed that ~4% (*n* = 12) of children had HbAS and single gene deletion alpha-thalassemia, but the prevalence of anemia did not differ among children with HbAS who did or did not have alpha-thalassemia in this small sample. A similar prevalence of HbS trait was observed among children recruited for a longitudinal study of HbS and malaria infection in southern Cameroon; the prevalence of HbAS was 22% among 156 children at birth and 13% in 84 older siblings (24–36 months). The same study also reported a lower prevalence of HbAS (2%) among individuals of the Bamiléké ethnic group compared to other groups, as we observed [35]. 

In multivariable models, we observed that Hb SS and homozygous α^+^thalassemia predicted Hb concentration, and HbAS and HbSS predicted anemia (with possibly a marginal effect of homozygous α^+^thalassemia). However, several other indicators representing nutritional status (micronutrient deficiencies) and infection and inflammation were also independent predictors. In exploratory calculations, the unadjusted (bivariate) population attributable fractions were 10.9% for inflammation (elevated CRP or AGP, possibly representing one or more infectious diseases), 6.5% for inflammation-adjusted iron deficiency, 5.3% for HbAS (sickle cell trait), 4.2% for malaria, 2.2% for homozygous α^+^thalassemia, and 1.9% for HbSS, although the cross-sectional study design does not permit causal attributions. An association between IHD and anemia in multivariable models was also reported in a survey in Kenya, although in that population anemia was associated with both heterozygous and homozygous α^+^thalassemia and not with Hb S [4,36]; in this survey, the population attributable fractions for IHD were also modest (<8%). These relationships underscore the multifactorial etiology of anemia and suggest that continued efforts to address both micronutrient deficiencies and infectious disease burden among children are necessary to reduce the prevalence of anemia among children in this setting. 

We used the cutoff of 110 g/L Hb to indicate anemia, for consistency with WHO recommendations. However, child age was positively associated with Hb concentrations, independent of IHD and indicators of nutritional status and infections. Other studies have also observed that Hb concentrations increase with age among children [37], suggesting that use of a single cutoff for children 6–59 months of age may overestimate the prevalence of anemia among younger children in this broad age bracket. Lower Hb cutoffs (<100 g/L) have been proposed for children at 9 mo, based on response to iron supplementation [38]; cutoffs for children older than 12 months of age may also need to be revisited. 

In this study population, α^+^thalassemia was not related to micronutrient status, consistent with a survey in Kenya, which found no relationship between α^+^thalassemia and iron status markers (following exclusion of children with inflammation)[15]. We observed that HbAS was associated with greater ferritin and RBP concentrations, after adjusting mathematically for markers of inflammation. Interestingly, Tsang et al. observed that individuals with homozygous α^+^thalassemia had lower prevalence of low RBP concentrations, although this relationship was no longer significant in multivariable models [16]. Possible mechanisms for relationships between IHD and micronutrient status, and their implications for health should be further explored. 

In this malaria-endemic setting, elevated sTfR was associated with markers of both iron deficiency and increased erythropoiesis (reticulocyte counts), even in a sample with a relatively low prevalence of malaria (8%). The extent to which sTfR reflects increased erythropoiesis, rather than iron deficiency, would likely be greater in populations with a greater malaria burden. In a previous survey in the northern regions of Cameroon, where the prevalence of current or recent malaria was ~30%, the difference between iron deficiency as measured by inflammation-adjusted ferritin compared to elevated sTfR was large (~35% based on low ferritin vs. ~85% based on elevated stfR)[28], possibly reflecting the effects of malaria-induced anemia on sTfR concentrations. Similar results have been observed elsewhere in sub-Saharan Africa [39,40]. Malaria infection was also associated with increased sTfR concentrations among young children in Burkina Faso [40], Cote d’Ivoire [41], and Kenya [19]. These results suggest that sTfR has limited specificity, and thus limited utility, as an iron status indicator in settings where malaria and other causes of increased erythropoiesis are prevalent without some other independent indicator of erythropoietic activity unrelated to iron status. In addition, consistent with the observed relationship between age and Hb, we also found negative associations between child age and sTfR concentrations. Similar relationships of sTfR with age have been observed in other settings, including the United States [42] and Greece [43]. Although it is possible that these relationships reflect higher risk of iron deficiency among younger children, the results suggest that sTfR, used alone, may overestimate the risk of iron deficiency among younger children. 

The sample size limits the precision of the estimates of the prevalence of anemia and IHD. In addition, although we assessed malaria, we did not attempt to identify other specific infections such as HIV or intestinal parasites; thus, we are unable to assess the extent to which these contribute to anemia. However, if these conditions are associated with elevated acute phase proteins, we can identify these individuals as having “anemia associated with inflammation”. In addition, these results may not represent the situation in other regions of the country, where the prevalences of anemia and conditions such as iron deficiency and inflammation were different [28]. 

A limitation of our cross-sectional data is that, where multiple potential causes of anemia are possible, we cannot determine which is responsible for insufficient Hb availability (or whether anemia may be due to a combination of factors). For example, in the primary analysis of this survey, improvements in iron status were noted among children in these two cities following fortification of wheat flour; however, their prevalence of anemia among children did not change compared to pre-fortification values [21]. Similarly, among children in Côte d’Ivoire, provision of iron-fortified complementary food to children 12–36 months of age for 9 months decreased the prevalence of ID (ferritin < 30 µg/L), but did not decrease the odds of anemia relative to the control group [44]. This suggests that removing a single risk factor for anemia may not be sufficient to reduce the prevalence of anemia, and the proportion of anemic individuals with concomitant ID may be greater than the proportion of iron deficient, anemic individuals who will respond to iron interventions. 

## 5. Conclusions

In sum, although IHD were prevalent, and HbS (both AS and SS) and homozygous α^+^thalassemia were associated with anemia, the impact was modest and the prevalence of anemia was high even among children without IHD. Efforts to address both micronutrient deficiencies and infectious disease are needed. Considering the multifactorial nature of anemia, nutrition program managers should be aware that some proportion of anemic subjects may not respond to interventions designed to address one or more causes of anemia (e.g., nutritional status or infectious disease), since anemia may reflect a combination of factors, underlying genetic conditions, or possibly inappropriate cutoffs (especially for young children). Because the relative proportions of modifiable and non-modifiable risk factors for anemia likely differ by setting, collection of setting-specific information on risk factors for anemia (with initial focus on the factors most likely to be common) should be prioritized. Finally, sTfR concentrations are not specific to iron deficiency in malaria-endemic areas and should be interpreted with caution.

## Figures and Tables

**Table 1:** Characteristics of children 12–59 months of age in Yaoundé and Douala, Cameroon ^1^.

Variable	Value
Age, months	32.8 ± 0.8
Male, *n* (%)	155 (50)
Breastfeeding, *n* (%)	11 (4)
Stunted (HAZ < −2), %	15.6 ± 2.4
Wasted (WHZ < −2), %	1.4 ± 0.7
Inflammation (CRP > 5 mg/L and/or AGP > 1 g/L), %	46.4 ± 3.1
Malaria, %	8.0 ± 2.1
Hemoglobin, g/L	110 ± 1
Anemia (Hb < 110 g/L), %	45.4 ± 2.9
Severe anemia (Hb < 70 g/L), %	1.0 ± 0.5
Reticulocytes, cells/µL	47,000 ± 2000
Reticulocytes, cells/µL ^2^	39,000 (29,000, 54,000) ^2^
Reticulocytes > 150,000 cells/µL, *n* (%)	5 (1.7)
Unadjusted ferritin, µg/L	51.1 ± 2.8
Adjusted ferritin ^3^, µg/L	32.2 ± 1.2
Adjusted Ferritin ^3^ < 12 µg/L, %	13.2 ± 2.0
sTfR, mg/L	8.22 ± 0.20
sTfR > 8.3 mg/L, %	25.4 ± 2.3
Unadjusted RBP, µmol/L	0.88 ± 0.02
Adjusted RBP ^3^, µmol/L	1.06 ± 0.01
Adjusted RBP ^3^ < 0.83 umol/L, %	13.5 ± 1.9
Plasma zinc, µg/dL	66.8 ± 1.6
Adjusted plasma zinc ^3^, µg/dL	74.0 ± 1.7
Low adjusted plasma zinc ^3,4^, %	23.2 ± 3.8
Folate, nmol/L	56.0 ± 2.1
Plasma B12, pmol/L	851 ± 51

^1^ Values are mean or % ± SE or *n* (%), unless otherwise indicated; *n* = 278–302, depending on the indicator. AGP, alpha-1-acid glycoprotein; CRP, C-reactive protein; HAZ, height-for-age *Z*-score; Hb, hemoglobin; sTfR, soluble transferrin receptor, WAZ, weight-for-age *Z*-score; WHZ, weight-for-height *Z*-score. ^2^ Median (25th percentile, 75th percentile). ^3^ Values adjusted for inflammation by regression analysis to values equivalent to those at CRP and AGP concentrations of 0.14 mg/L CRP and 0.52 g/L AGP (the 10th percentile among individuals with CRP < 5 and AGP < 1). ^4^ Low plasma zinc defined as <65 µg/dL for morning samples and <57 µg/dL for afternoon samples [23], after adjusting for inflammation as noted above.

**Table 2:** Prevalence of inherited hemoglobin disorders among a representative sample of children 12–59 months of age in Yaoundé and Douala, Cameroon ^1^.

		Urban Total	Yaoundé	Douala
Any Hb S or alpha-globin ^2^ deletion		42.9 (37.4–48.4)	48.9 (39.7–58.1)	37.1 (30.1–44.0)
Sickle cell disease or trait ^3^				
	HbSS	1.6 (0.2–3.0)	2.7 (0.1–5.3)	0.6 (0–1.9)
	HbAS	13.7 (9.7–17.8)	12.6 (6.3–18.9)	14.8 (9.1–20.5)
α^+^-thalassemia ^2^				
	Homozygous (–α/–α)	3.1 (1.1–5.2)	4.9 (1.2–8.6)	1.4 (0–3.5)
	Heterozygous (–α/αα)	30.6 (25.4–35.8)	37.0 (27.7–46.4)	24.4 (18.9–29.9)
Other Hb conditions ^3^				
	Increased F	4.7 (2.7–6.6)	5.5 (2.5–8.4)	3.9 (1.2–6.7)

^1^ Values are the mean (95% CI). Hb, hemoglobin. ^2^ *n* = 288. ^3^ *n* = 295.

**Table 3:** Simple and multiple regression models of predictors of Hb concentration and anemia ^1^.

Independent Variables	Dependent Variable: Hb Concentration (g/L^2^)				Dependent Variable: Anemia (Hb < 110 g/L)			
	Bivariate Relationships		Adjusted Model		Bivariate Relationships		Adjusted Model	
	β (SE)	*p*	β (SE)	*p* ^2^	OR (95% CI)	*p*	OR (95% CI)	*p* ^2^
Age, months	25.4 (14.4)	0.088	24.7 (11.0)	0.033	0.972 (0.949–0.997	0.026	0.963 (0.938–0.990)	0.007
Male	−598 (331)	0.080	--	0.25	0.76 (0.45–1.28)	0.30	--	0.48
Hb AS	−138 (368)	0.71	--	0.15	2.01 (1.01–4.02)	0.047	2.14 (1.13–4.07)	0.020
Hb SS	−6960 (1020)	<0.0001	−6790 (880)	<0.0001	>999 (>999–>999) ^5^	<0.0001	>999 (>999–>999)	<0.001
α^+^thalassemia heterozygote	−149 (341)	0.67	--	0.31	0.81 (0.46–1.42)	0.47	--	0.71
α^+^thalassemia homozygote	−1470 (490)	0.006	−1270 (440)	0.007	4.31 (1.03–17.93)	0.045	--	0.11
Malaria	−2050 (690)	0.006	−1760 (620)	0.008	2.62 (1.10–6.27)	0.030	2.80 (1.03–7.64)	0.045
Adjusted RBP ^3^ < 0.83 µmol/L	−1360 (430)	0.004	−620 (299)	0.048	2.00 (1.09–3.67)	0.026	--	0.13
Adjusted ferritin ^3^ < 12 µg/L	−1270 (310)	0.0003	−1170 (330)	0.001	2.46 (1.38–4.41)	0.002	2.08 (1.03–4.17)	0.040
Low adjusted plasma zinc ^3,4^	−425 (399)	0.30	--	0.99	1.26 (0.68–2.32)	0.46	--	0.90
Plasma CRP, mg/L	−334 (129)	0.015	--	0.71	1.05 (1.01–1.09)	0.015	1.05 (1.01–1.09)	0.014
Plasma AGP, g/L	−1520 (550)	0.010	−1290 (470)	0.010	2.46 (1.16–5.21)	0.019	--	0.44
Plasma folate, nmol/L	18.4 (8.1)	0.03	--	0.27	0.975 (0.962–0.989)	0.0003	0.976 (0.961–0.991)	0.002
Plasma vitamin B12, pmol/L	1.45 (0.38)	0.0007	0.984 (0.312)	0.004	0.999 (0.998–1.000)	0.012	--	0.078

^1^ Analysis conducted using SAS PROC SURVEYREG and SURVEYLOGISTIC, for hemoglobin concentration (*n* = 266; *R*^2^ = 0.34 for final model) and anemia (*n* = 273 for final model), respectively. AGP, α_1_-acid glycoprotein; CRP, C-reactive protein; Hb, hemoglobin; OR, odds ratio. ^2^ For variables removed from the model, *p*-values indicate the adjusted *p*-value at the step at which the variable was removed from the model. For variables retained in the final model, the *p*-value reflects the final adjusted model (with all non-significant variables removed). ^3^ Values adjusted for inflammation by regression analysis to values equivalent to those at CRP and AGP concentrations of 0.14 mg/L CRP and 0.52 g/L AGP (the 10th percentile among individuals with CRP < 5 and AGP < 1). ^4^ Low plasma zinc defined as <65 µg/dL for AM samples and < 57 µg/dL for PM samples [23], after adjusting for inflammation as noted above. ^5^ All children with the HbSS genotype were anemic.

**Table 4:** Characteristics associated with elevated sTfR among children 12–59 months of age in Yaoundé and Douala, Cameroon.

	sTfR ≤ 8.3 mg/L	sTfR > 8.3 mg/L	*p*
*n*	211–221	72–76	
Age, months	34.3 ± 1.0	29.9 ± 1.5	0.014
Male, %	46.6 ± 3.2	64.1 ± 5.9	0.012
Hemoglobin, g/L	112 ± 1	102 ± 2	<0.001
Anemic, %	37.3 ± 3.1	69.4 ± 4.5	<0.001
Malaria, %	5.1 ± 1.7	18.9 ± 5.5	0.0005
Reticulocytes, cells/nL	38.8 ± 1.6	68.4 ± 6.3	<0.001
Reticulocytes > 150 cells/nL, %	0	6.8 ± 2.9	<0.001
Inflammation (CRP > 5 mg/L and/or AGP > 1 g/L)	42.1 ± 3.1	59.3 ± 5.6	0.002
CRP, mg/L	4.23 ± 0.49	5.31 ± 1.01	0.31
AGP, mg/L	0.94 ± 0.02	1.06 ± 0.04	0.003
Adjusted ferritin ^2^ < 12 µg/dL, %	6.3 ± 1.6	33.5 ± 6.8	<0.001
Adjusted RBP ^2^ < 0.83 µmol/L, %	11.7 ± 2.2	18.5 ± 4.7	0.18
Low adjusted zinc ^2,3^, %	21.5 ± 3.8	25.8 ± 5.8	0.38
Plasma folate, nmol/L	56.7 ± 2.4	54.3 ± 2.7	0.44
Plasma vitamin B12, pmol/L	828 ± 30	778 ± 84	0.54
HbAS genotype	13.0 ± 2.1	16.2 ± 4.8	0.52
HbSS genotype	0	6.3 ± 2.7	<0.001
α^+^thalassemia heterozygote	31.8 ± 2.9	27.7 ± 4.5	0.44
α^+^thalassemia homozygote	2.4 ± 1.0	5.4 ± 2.4	0.17

^1^ Values are mean or % ± SE. AGP, alpha-1-acid glycoprotein; BIS, body iron stores; CRP, C-reactive protein; Hb, hemoglobin; RBP, retinol-binding protein; sTfR, soluble transferrin receptor. ^2^ Values adjusted for inflammation by regression analysis to values equivalent to those at CRP and AGP concentrations of 0.14 mg/L CRP and 0.52 g/L AGP (the 10th percentile among individuals with CRP < 5 and AGP < 1). ^3^ Low plasma zinc defined as <65 µg/dL for AM samples and <57 µg/dL for PM samples [23], after adjusting for inflammation as noted above.
